# Exploring the mechanism of action of Sparganii Rhizoma-Curcumae Rhizoma for in treating castration-resistant prostate cancer: a network-based pharmacology and experimental validation study

**DOI:** 10.1038/s41598-024-53699-5

**Published:** 2024-02-07

**Authors:** Litong Wu, Haijun Chen, Yan Long, Junfeng Qiu, Xinjun Dai, Xujun You, Tiantian Li

**Affiliations:** 1 The First Clinical College of Traditional Chinese Medicine, Hunan University of Chinese Medicine, Changsha, 410208 People’s Republic of China; 2 School of Pharmaceutical Sciences, Hunan University of Medicine, Huaihua, 418000 People’s Republic of China; 3 Shenzhen Traditional Chinese Medicine Hospital, Shen zhen, 518033 People’s Republic of China; 4 Liuyang Hospital of Traditional Chinese Medicine Affiliated to Hunan University of Chinese Medicine, Changsha, 410300 People’s Republic of China; 5 Department of Andrology, Shenzhen Bao’an Traditional Chinese Medicine Hospital Group, Guangzhou University of Chinese Medicine, Shenzhen, 518100 People’s Republic of China; 6 Department of Otorhinolaryngology, Shenzhen Bao’an Traditional Chinese Medicine Hospital Group, Guangzhou University of Chinese Medicine, Shenzhen, 518100 People’s Republic of China

**Keywords:** Cancer, Cell biology, Computational biology and bioinformatics, Oncology, Urology

## Abstract

Sparganii Rhizoma-Curcumae Rhizoma (SR-CR) is a classic drug pair for the treatment of castration-resistant prostate cancer (CRPC), but its mechanism has not been clarified. The study aims to elucidate the potential mechanism of SR-CR in the management of CRPC. The present study employed the TCMSP as well as the SwissTargetPrediction platform to retrieve the chemical composition and targets of SR-CR. The therapeutic targets of CRPC were identified through screening the GeneCards, Disgenet, and OMIM databases. Subsequently, the Venny online platform was utilized to identify the shared targets between the SR-CR and CRPC. The shared targets were enrichment analysis using the Bioconductor and Kyoto encyclopedia of genes and genomes (KEGG) databases. The active ingredients and core targets were verified through molecular docking and were validated using PC3 cells in the experimental validation phase. A total of 7 active ingredients and 1126 disease targets were screened from SR-CR, leading to a total of 59 shared targets. Gene Ontology (GO) analysis resulted in 1309 GO entries. KEGG pathways analysis yielded 121 pathways, primarily involving cancer-related signaling pathways. The results from molecular docking revealed stable binding interactions between the core ingredients and the core targets. In vitro cellular assays further demonstrated that SR-CR effectively suppressed the activation of the Prostate cancer signaling pathway in PC3 cells, leading to the inhibition of cell proliferation and promotion of apoptosis. The SR-CR exert therapeutic effects on CRPC by inhibiting cell proliferation and promoting apoptosis through the Prostate cancer signaling pathway.

## Introduction

Prostate cancer is a prevalent urogenital malignant, with the highest incidence and mortality rates in the United States, according to the latest statistics from the international topical journal CA 2022^1^. The prevalence of early screening in China has made prostate cancer the fastest-growing malignancy in the last decade, posing a significant public health problem that seriously threatens men's health^2^. Castration-resistant prostate cancer (CRPC) is a highly metastatic and drug-resistant form of advanced prostate cancer that is the main cause of decreased quality of life and short survival in patients^3^. Although second-generation anti-androgen therapeutic agents have significantly improved the survival rate of CRPC patients, a considerable proportion of them still fail to achieve complete remission^4^. Thus, there is an urgent need to explore new effective means of treating CRPC to alleviate patients' suffering and improve their quality of life. Chinese medicine has become an integral part of the comprehensive treatment of CRPC in recent years, offering various benefits, such as supporting and fighting cancer, regulating the body's constitution, alleviating the adverse effects caused by chemotherapy, and improving the quality of life of patients^5^. *Sparganii Rhizoma-Curcumae Rhizoma* (SR-CR) is a classical and effective anti-tumor drug pair in Chinese medicine, consisting of *Sparganium stoleniferum* Buch.—Ham. and Curcuma longa or Curcuma phaeocaolis Val., Curcuma kwangsiensis S.G. Lee et C.F. Liang, or Curcuma wenyuyin Y.H. Chen et C. Ling. Modern pharmacological studies have confirmed the pharmacological effects of these constituents, such as anti-tumor, anti-platelet aggregation, anti-thrombotic, improving blood rheology, analgesia, cardiovascular regulation, anti-tissue fibrosis, anti-inflammatory, antibacterial and antiviral, hypoglycemic, antioxidant, and anti-mammary gland hyperplasia effects^6,7^.

**Figure 1:**
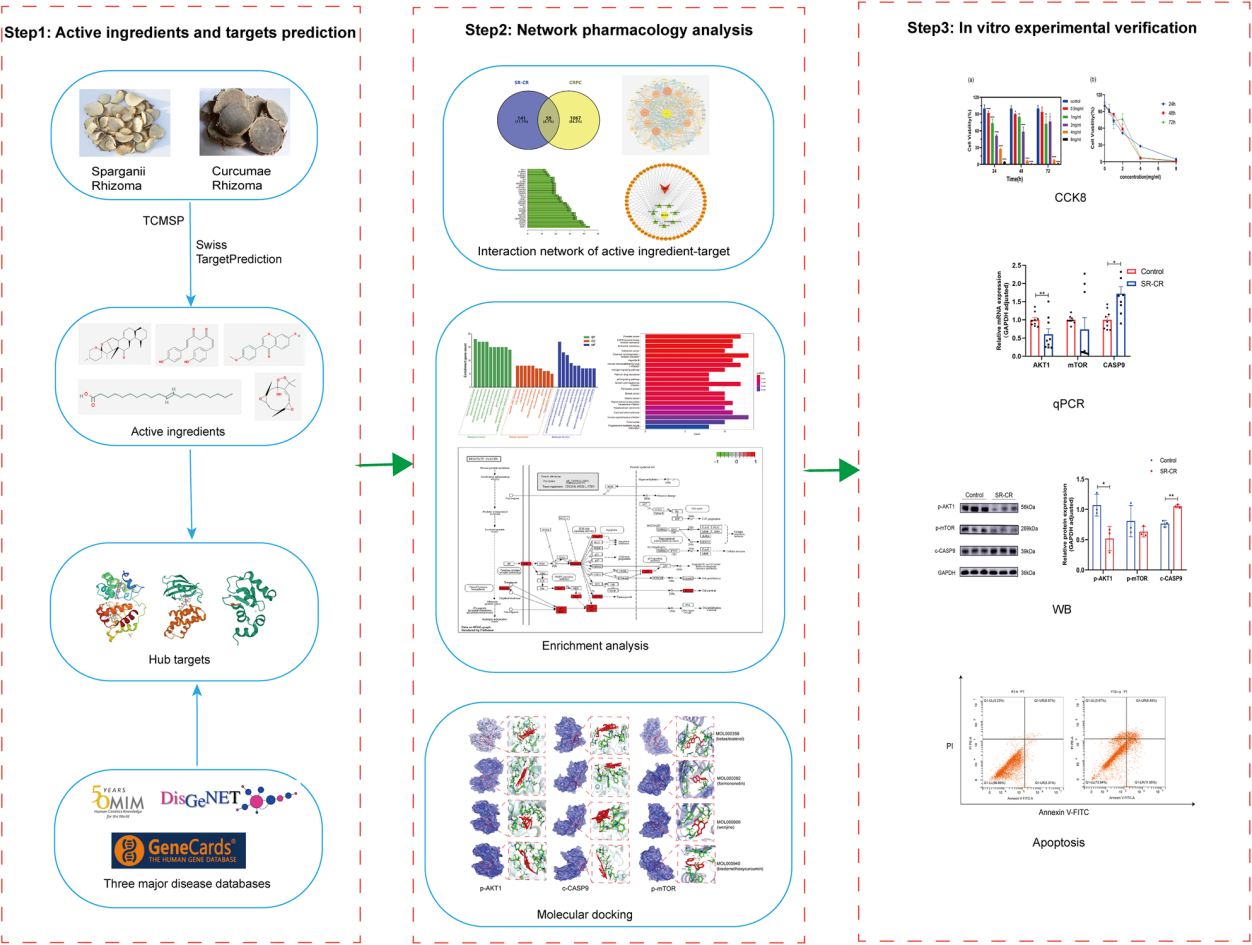
The process of this research.

Combining SR-CR drug pair has a theoretical basis and certain combination methods that have been clinically proven effective in reducing toxicity and producing synergistic effects. Modern pharmacological studies have confirmed that the combination of Chinese medicine pairs generally has the pharmacological properties of multi-component, multi-target, and synergistic effects^8^. In the study, we applied a network pharmacology approach to predicting the new potential targets of SR-CR drug pair for treating CRPC and used in vitro cellular experiments to validate the molecular mechanisms of the predicted active ingredients and key pathways. This study provides a reliable basis for further understanding of the new potential mechanism of action of SR-CR drug pair in the treatment of CRPC (Fig. 1).

## Results

### Identification of active ingredients and potential targets of SR-CR

Using a combination of the TCMSP database and literature search, we identified four active ingredients of Trigonella and three active ingredients of Allium for SR-CR (Table 1). We obtained a total of 119 potential therapeutic targets for Trigonella active ingredients and 81 potential therapeutic targets for Curcuma longa active ingredients after prediction and screening of the TCMSP database and Swiss TargetPrediction database, followed by UniProt database normalization and removal of duplicate targets.

**Table 1:** The main active ingredients of SR-CR.

Ingredient number	Name	OB%	DL
MOL000296	Hederagenin	36.91	0.75
MOL000358	Beta-sitosterol	36.91	0.75
MOL000392	Formononetin	69.67	0.21
MOL000449	Stigmasterol	43.83	0.76
MOL000906	Wenjine	47.93	0.27
MOL000940	Bisdemethoxycurcumin	77.38	0.26
MOL001297	Trans-gondoic acid	30.7	0.2

### Identification of potential therapeutic targets for CRPC

**Figure 2:**
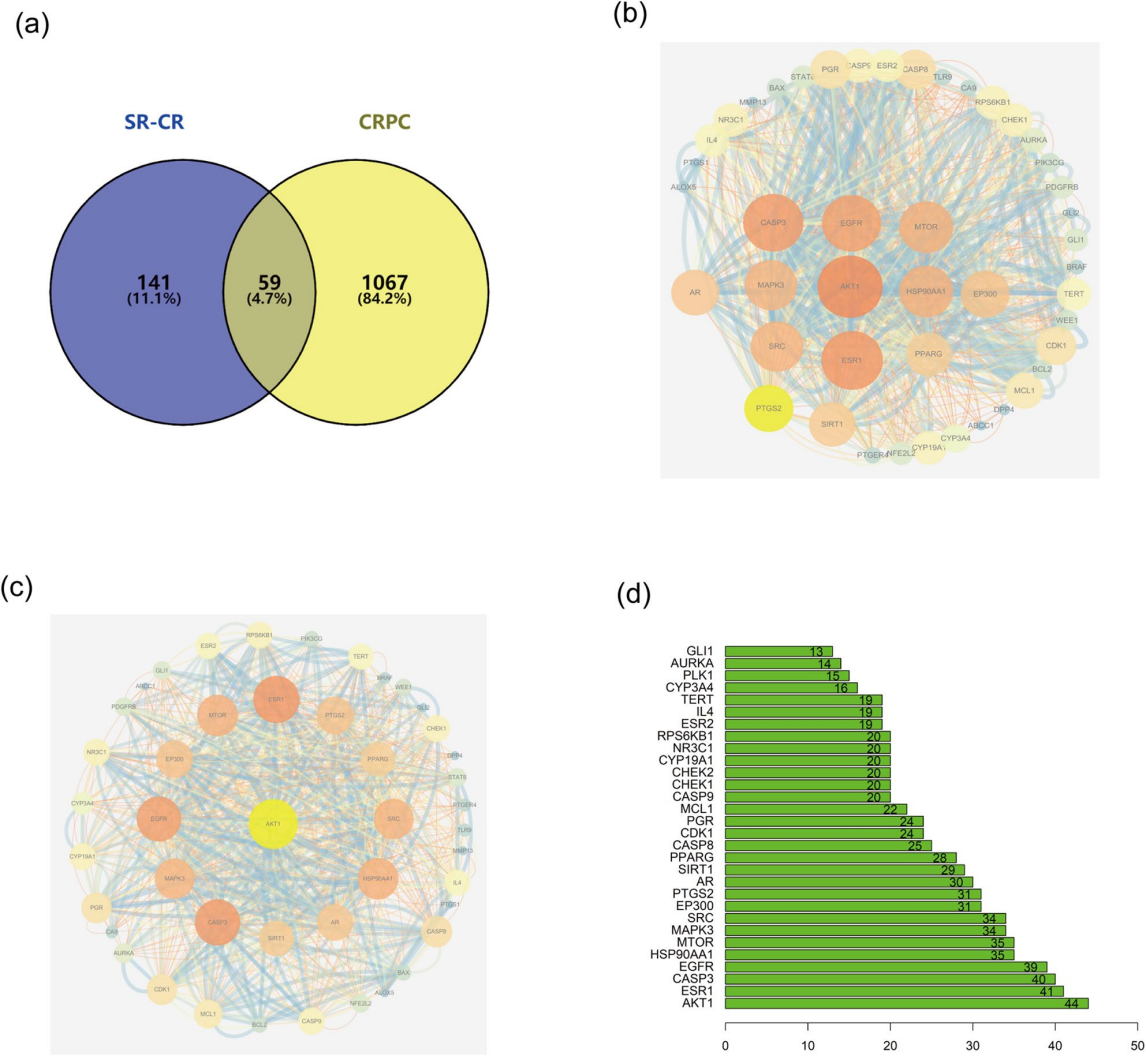
(**a**) Shared targets of SR-CR and CRPC. (**b**) Interaction network diagram of shared target proteins. (**c**) Interaction network diagram of target proteins with AKT1 as the core. (**d**) Interaction frequency bar diagram of shared targets.

We obtained 1126 potential therapeutic targets for CRPC from GeneCards, Disgenet, and OMIM databases. After comparing these targets with the potential targets of SR-CR, we identified 59 targets that were specific to CRPC (Fig. 2a). The network diagram of the interrelationship between these 59 targets was constructed using the Strings database and visualized using Cytoscape (Fig. 2b). The top-ranked target, AKT1, was used as the core for network association among the targets (Fig. 2c). The count.R plugin in the R programming software was used to tally the connection frequencies of the common targets, and the top 30 protein targets were visually displayed (Fig. 2d). The top 10 core proteins were identified as AKT1, ESR1, CASP3, EGFR, HSP90AA1, mTOR, MAPK3, SRC, EP300, and PTGS2.

### Construction of active ingredient-target-disease network

**Figure 3:**
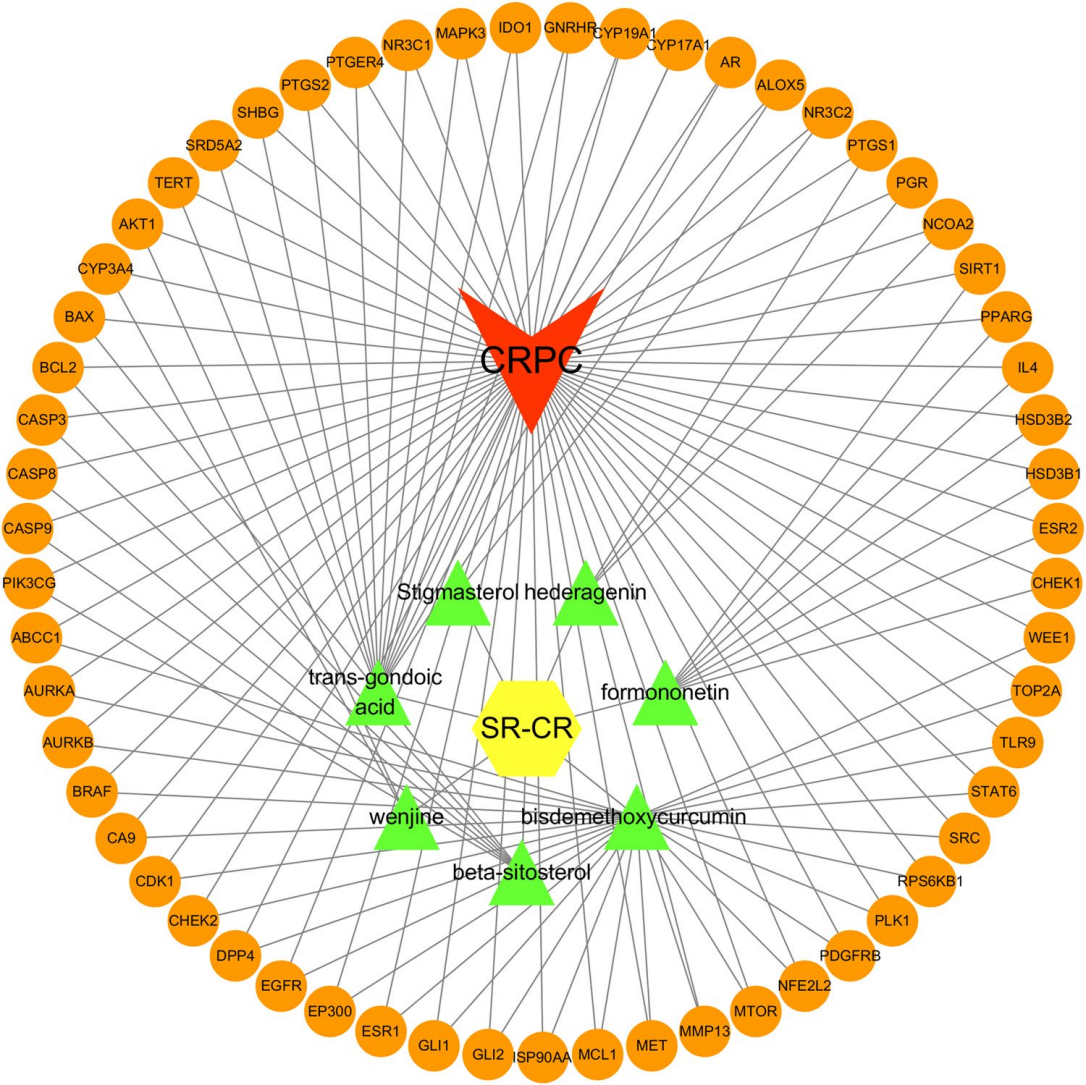
Drug-active ingredient-shared target-disease network diagram of SR-CR for the treatment of CRPC.

Using Cytoscape software, we constructed an active ingredient-shared target-disease network to visualize the potential mechanism of action of the SR-CR drug pair for the treatment of CRPC (Fig. 3). The network consisted of drug-active ingredient-shared target-disease nodes, with orange circle representing shared targets, yellow hexagon representing the trigone-curcumin pair, green triangle representing the active ingredient, and red quadrangle representing CRPC. The active ingredients included formononetin, beta-sitosterol, wenjine, hederagenin, and Stigmasterol.

### GO enrichment analysis and KEGG pathway enrichment analysis

**Figure 4:**
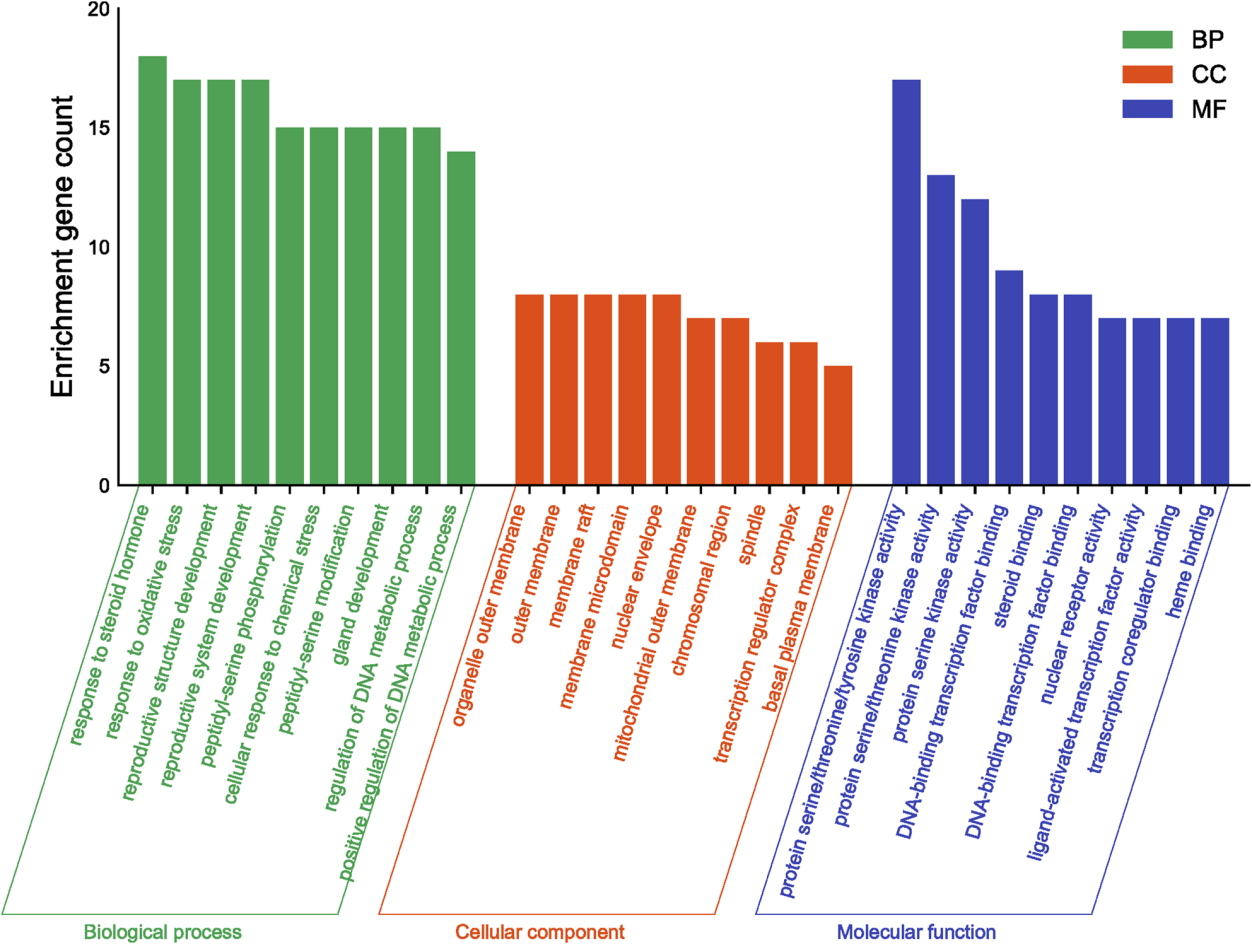
Histogram of GO analysis of SR-CR for CRPC.

**Figure 5:**
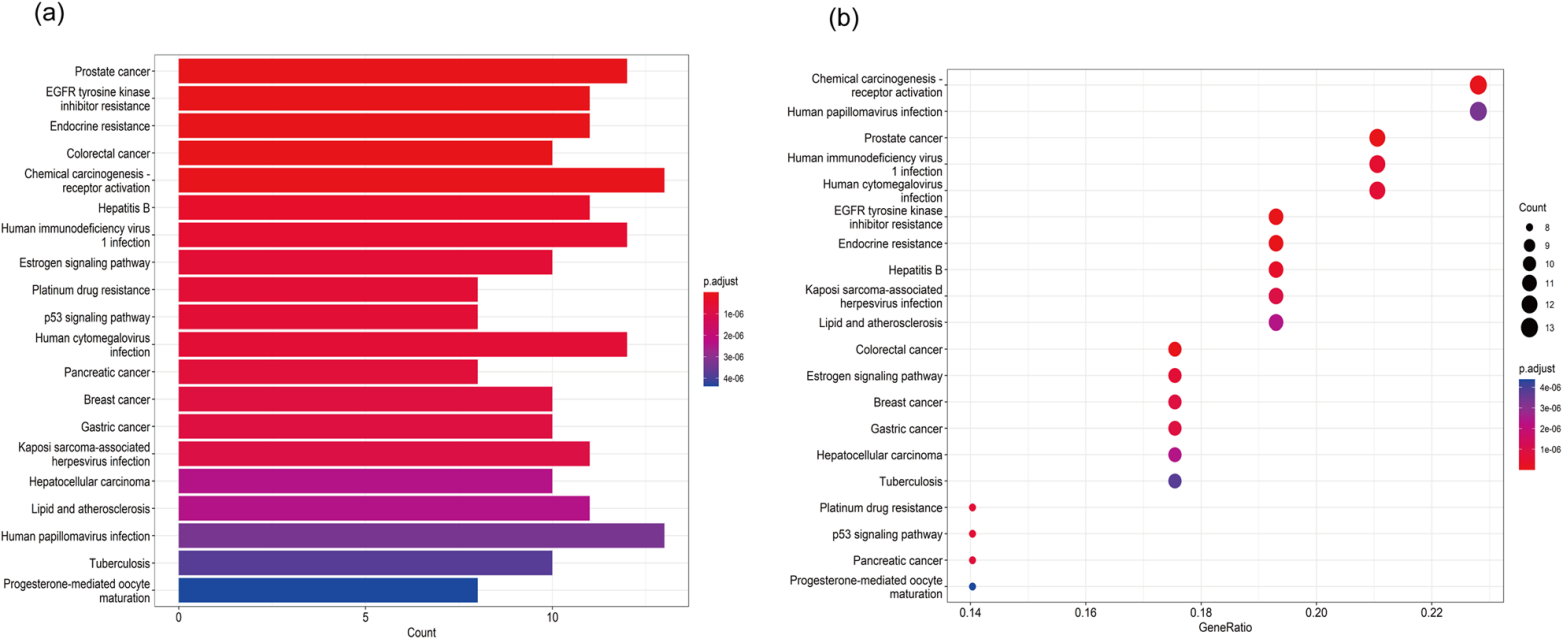
(**a**) KEGG histogram of SR-CR for CRPC. (**b**) KEGG bubble plot of SR-CR for CRPC.

**Figure 6:**
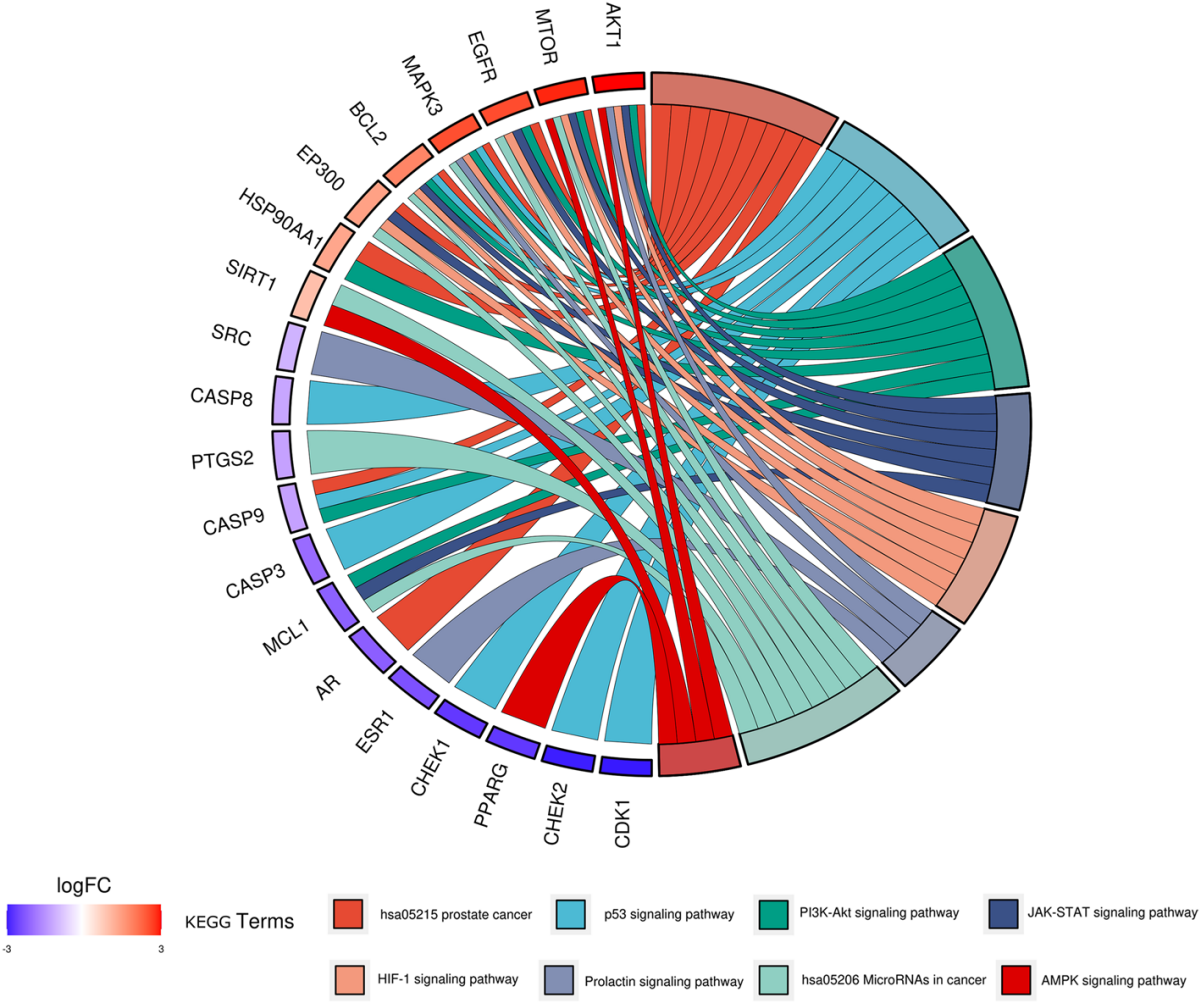
KEGG enrichment analysis string diagram (different colors on the right side of the diagram represent different signaling pathways, and the number of bands represents the number of associated genes) (1 bar on the left side represents 1 gene, the redder the color, the larger the logFC value).

**Figure 7:**
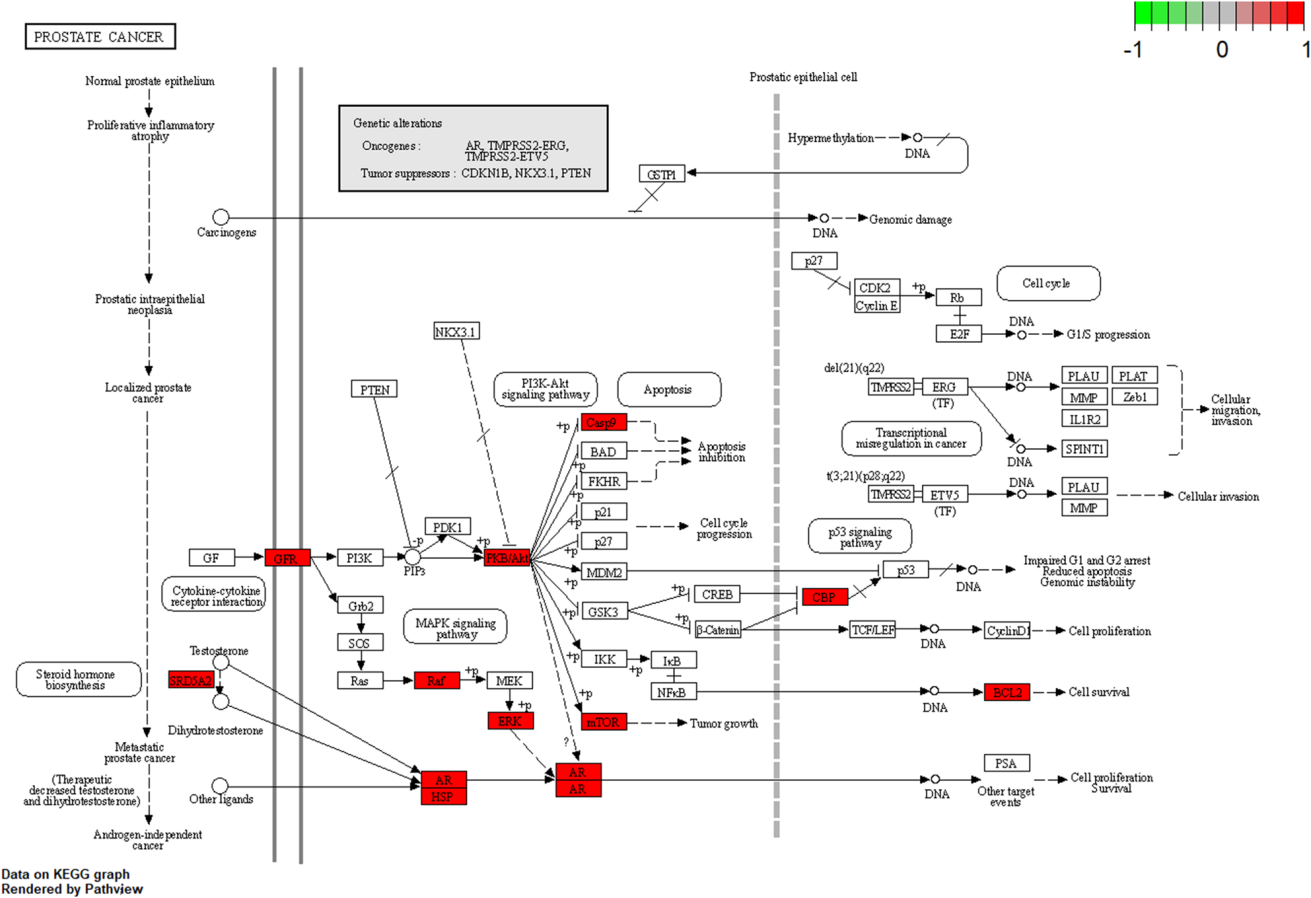
Potential targets and mechanisms of action of SR-CR in Prostate cancer signaling pathway.

Using the Bioconductor bioinformatics package in R language software, we performed GO and KEGG analysis to identify the biological processes, cellular components, molecular functions, and signaling pathways involved in the anti-cancer effects of SR-CR. The 1309 GO entries obtained included 1194 biological process entries, 35 cellular component entries, and 80 molecular function entries. The top 10 entries in each category were taken for visual analysis (Fig. 4). The KEGG enrichment identified 121 pathways, with the top 20 pathways displayed using bar graphs and bubble graphs (Fig. 5). The eight KEGG pathways related to the development of prostate cancer resistance were also analyzed (Fig. 6). AKT1, mTOR, CASP9, and BCL2 were identified as the top targets involved in the anti-prostate cancer resistance effect of the SR-CR drug pair (Fig. 7).

### Validation of molecular docking

**Figure 8:**
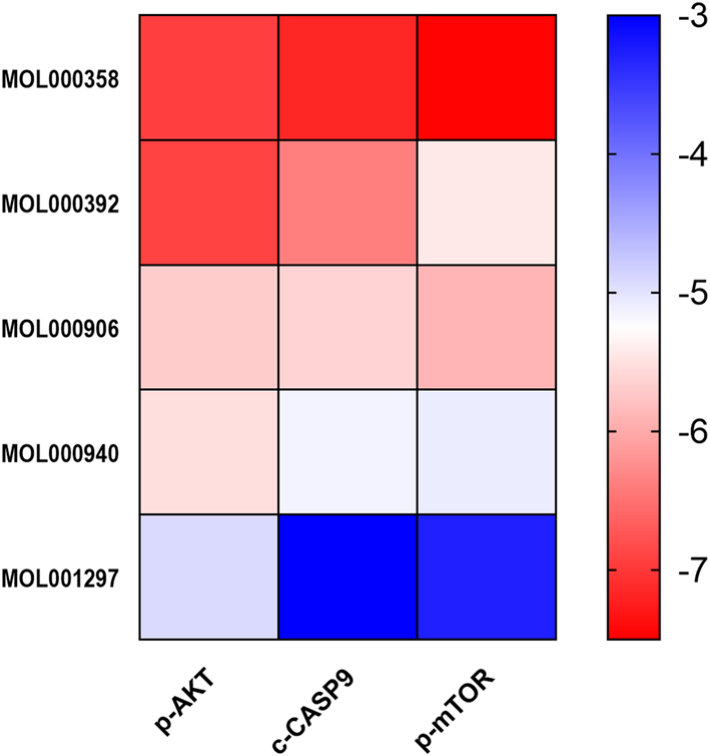
Binding energy thermogram of the five core active ingredients in SR-CR with the three core targets in CRPC.

**Figure 9:**
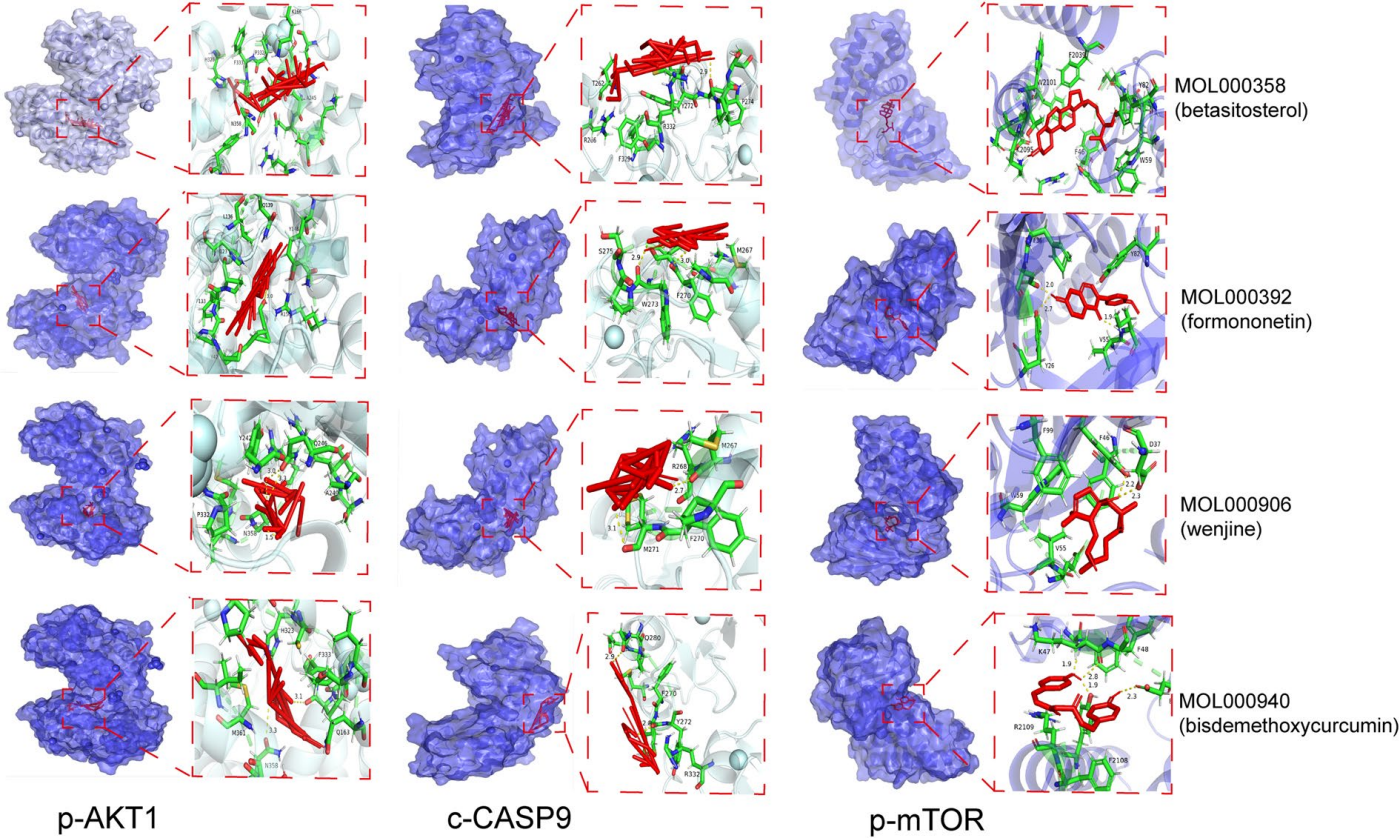
Docking pattern of the four core active ingredients in SR-CR with the three core targets in CRPC.

Using the PDB protein database, we obtained core target information for CRPC and performed molecular docking of the core active ingredients with the core targets to determine ligand-receptor docking binding energy (Table 2 and Fig. 8). Beta-sitosterol, formononetin, wenjine, and bisdemethoxycurcumin showed good docking activity with the core targets of CRPC, AKT1, mTOR, and CASP9 (Fig. 9).

**Table 2:** Information of 3 proteins involved in molecular docking.

Target	Uniprot-ID	PDB-ID	Ligand-ID
AKT1	P31749	1H1O	GOL
mTOR	P42345	1NSG	RAD
CASP9	P55211	3D9T	ZN

### Cell viability assay

**Figure 10:**
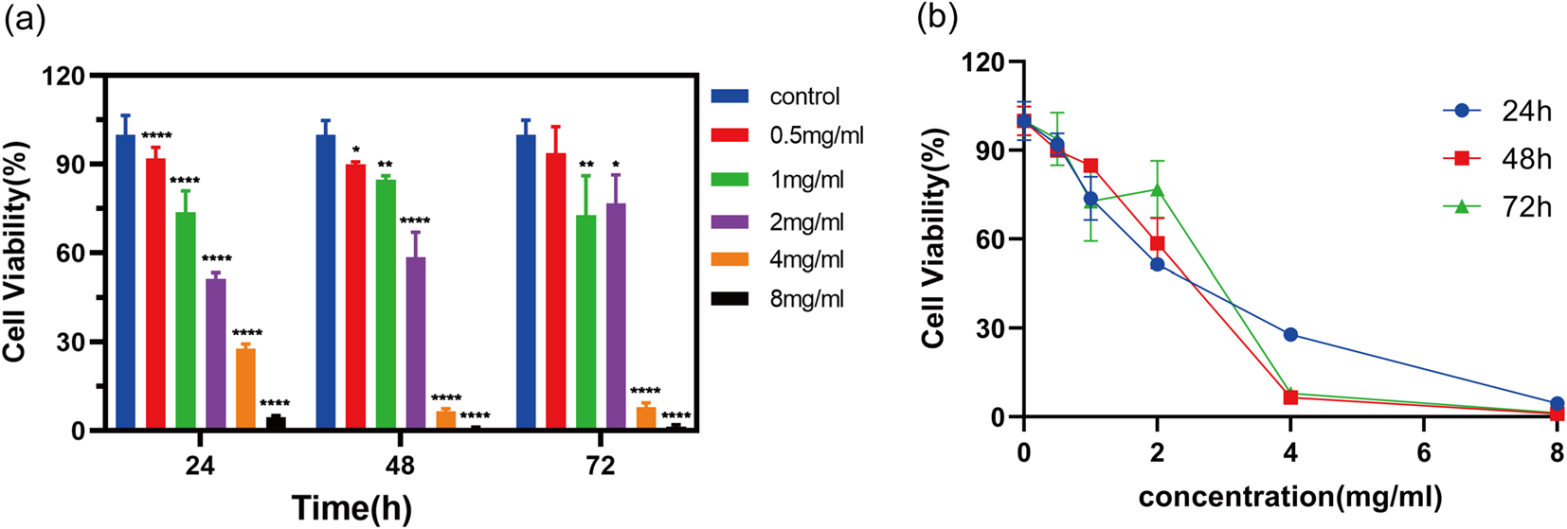
(**a**) Bar chart of viability rate of PC3 cells treated with different concentrations of SR-CR intervention at three time points (Compared with the Control group, *P < 0.05, **P < 0.01, ****P < 0.0001). (**b**) Line chart of Viability rate of PC3 cells treated with different concentrations of SR-CR intervention at three time points.

The effect of SR-CR pairs on PC3 cells was assessed using a cell viability assay after 24, 48, and 72 h of treatment. The result showed that SR-CR exhibited a concentration-dependent proliferation inhibition compared to the control group (*P* < 0.05), with IC_50_ values of 3.32 mg/mL, 2.31 mg/mL, and 2.83 mg/mL, respectively, as shown in Fig. 10. Microscopic observations (40x) revealed that the growth rate of control PC3 cells was faster after 48 h, and therefore, the IC_50_ of SR-CR at 48 h was selected for subsequent experiments.

### Analysis of apoptosis detection

**Figure 11:**
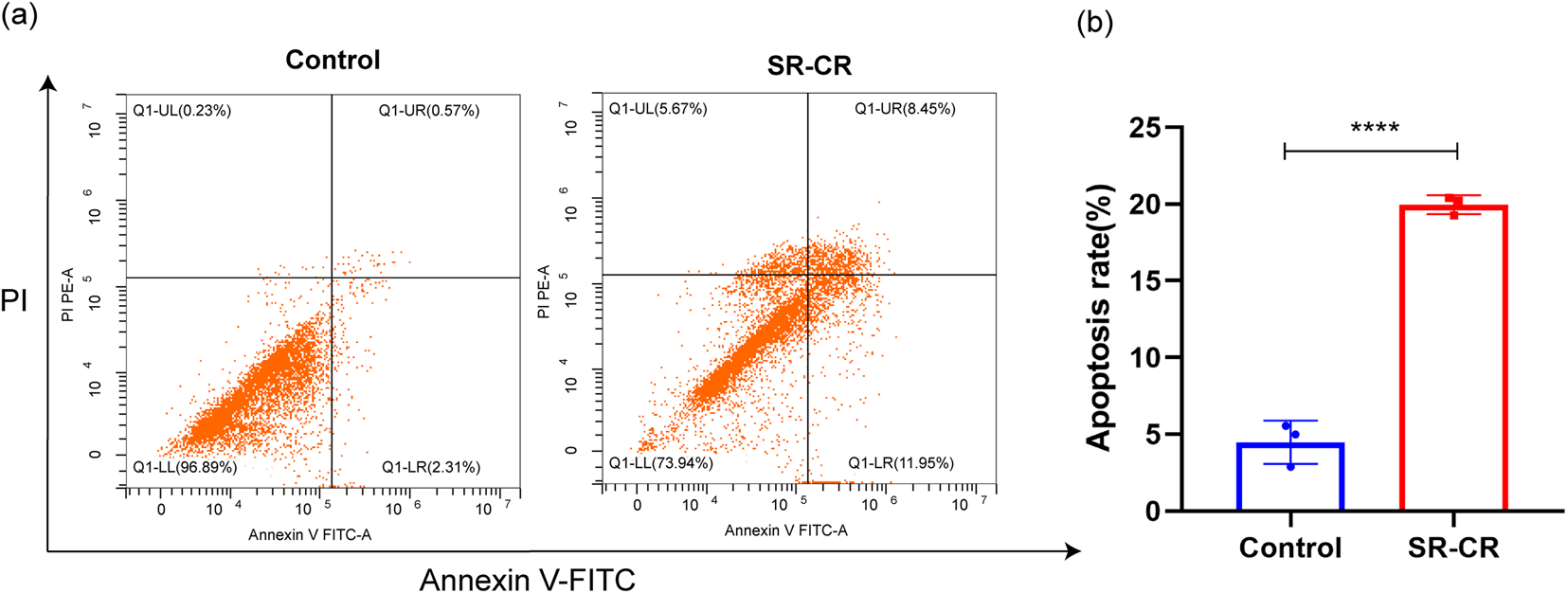
(**a**) Grid diagram shows the effect of SR-CR on PC3 cells apoptosis. (**b**) Bar chart shows the effect of SR-CR on PC3 cell apoptosis (Note: LL is live cells, LR early apoptotic cells, UR are late apoptotic and dead cells, UL are debris and damaged cells, conventional apoptosis rate can be calculated as UR + LR; compared with the Control group, ****P < 0.0001).

The apoptosis of PC3 cells after SR-CR intervention was assessed using flow cytometry. The results showed that the apoptosis rate in the SR-CR group (19.95 ± 0.62)% was significantly higher than the control group (4.48 ± 1.41)% (*P* < 0.05) (Fig. 11). Microscopic observations (400x) revealed that the SR-CR-treated cells exhibited reduced number, smaller in size, crinkled morphology, and increased cell rupture, suggesting that one of the mechanisms of SR-CR anti-CRPC action may be mediated through apoptosis of tumor cells.

### Differential gene analysis

**Figure 12:**
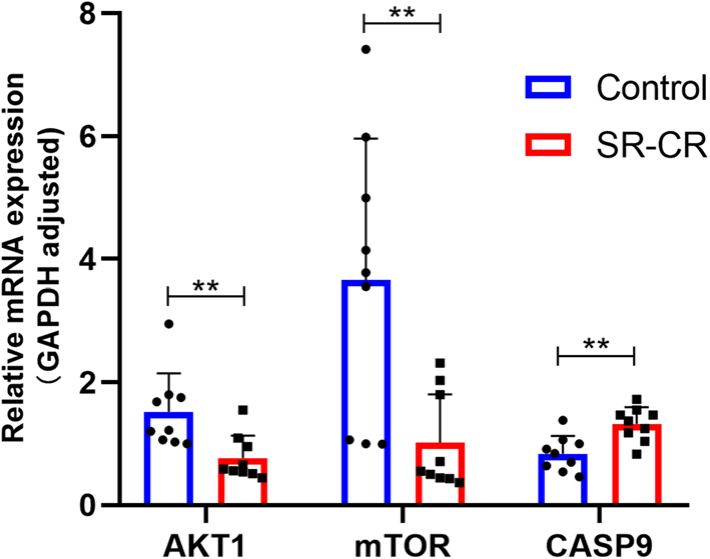
Effect of SR-CR on mRNA expression of core targets in PC3 cells (Compared with the Control group, **P < 0.05).

The expression of mRNA of core targets after the action of the SR-CR drug pair on PC3 cells was detected using the qPCR method. The results showed that compared to the control group, the mRNA expression of CASP9 (1.32 ± 0.27)% was significantly increased in the SR-CR group (2.31 mg/mL), while the mRNA expression of AKT1 (0.76 ± 0.36)% was significantly decreased, and the differences were statistically significant (*P* < 0.05). The mRNA expression of mTOR (1.02 ± 0.79)% was decreased, and the differences were statistically significant (*P* < 0.05) (Fig. 12).

### Differential protein analysis

**Figure 13:**
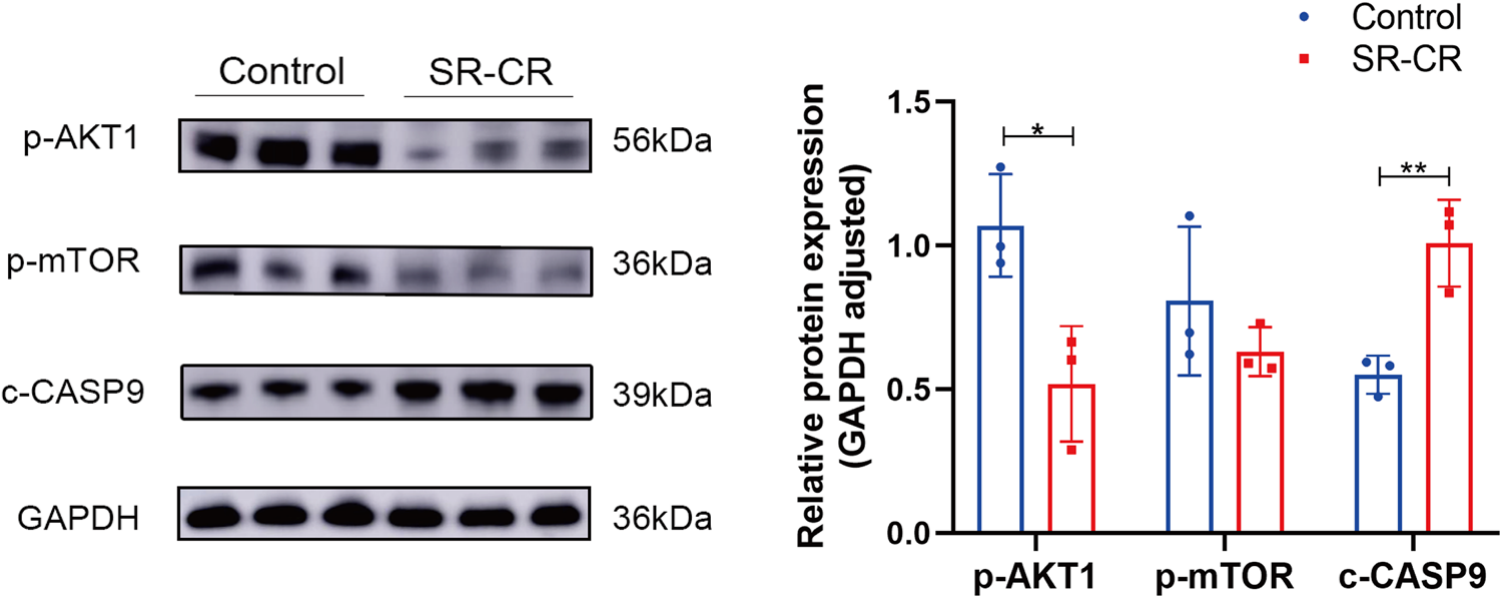
Effect of SR-CR on the expression of core target proteins in PC3 cells (Compared with the Control group, *P < 0.05, **P < 0.01).

The expression of proteins related to the prostate cancer signaling pathway was detected using western blot assay to verify the core signaling pathway obtained by enrichment of the KEGG pathway. The expression levels of p-AKT1 and p-mTOR were reduced in the SR-CR group, with p-AKT1 showing a statistically significant reduction (*P* < 0.05), while the expression level of c-CASP9 was significantly increased (*P* < 0.05) (Fig. 13). These results suggest that the prostate cancer signaling pathway may play a role in the anti-CRPC action of SR-CR.

## Materials and methods

### Screening of active ingredients and targets of SR-CR drug pair

The identification of active constituents in Sanling-Curcuma was performed using the Chinese Medicine Systematic Pharmacology Data and Analysis Platform (TCMSP, https://old.tcmsp-e.com/tcmsp.php), with "Sanling" and "Curcuma" used as search keywords, respectively. The active ingredients of the drug pair were identified by searching "san leng" and "zedoary" as keywords. To screen for potentially effective active ingredients, criteria were set based on their absorption, distribution metabolism, and excretion (ADME) characteristics in vivo, with a requirement for oral bioavailability (OB) ≥ 30% and drug-like properties ≥ 0.18^9^. Subsequently, the potential therapeutic targets of the screened active ingredients were obtained using the TCMSP platform, SwissTargetPrediction (http://www.swisstargetprediction.ch/) platform, and the UniProt database (https://www.uniprot.org) with normalization being performed. The UniProt database was chosen for protein name standardisation because it integrates three major databases, Swiss Prot, TrEMBL and PIR-PSD, and is the most informative and resourceful protein database.

### CRPC action target screening

The present study employs the keyword "CRPC" to identify therapeutic targets for CRPC from three major disease databases: GeneCards database (https://www.genecards.org/), Disgenet database (https://www.disgenet.org/), and OMIM database (https://omim.org/). To eliminate duplicate genes, the screening process was conducted in a rigorous manner. Furthermore, the UniProt database was used as a standardisation of disease target protein names.

### Acquisition of shared targets and PPI network construction

The putative therapeutic targets of the active constituents of the SR-CR pair were subjected to analysis using the Venny 2.1.0 platform (https://bioinfogp.cnb.csic.es/tools/venny/index.html), in conjunction with the CRPC disease targets, to identify the common targets and generate the corresponding Venny diagram. Subsequently, the common targets were mapped onto the STRING database with “Homo sapiens” specified as the organism. A "medium confidence" threshold and "combined score > 0.4" were applied to construct the protein–protein interaction (PPI) network diagram. The STRING database is a database that searches for known interactions between proteins and predicted interactions between proteins. Studying the interaction network between proteins helps to uncover the core regulatory genes. There are already many databases of protein interactions, and STRING is one of them that covers the most species and has the largest interaction information. In the constructed PPI network, the Degree value of each target was represented by its node size and color, with larger nodes and redder colors indicating higher Degree values and greater centrality within the network. The top five proteins were identified as the core targets of SR-CR against CRPC, following filtration using the MCODE plug-in in Cytoscape (https://cytoscape.org/).

### Construction of component-disease-target network

The relationship between the drug, active ingredient, disease, and the common target was established as nodes using an Excel table and imported into Cytoscape 3.7.2 software to construct a network diagram of Chinese medicine—ingredient—disease—target. The Network Analyzer plug-in in Cytoscape (http://www.cytoscape.org/) was utilized to perform topology analysis, which involved calculating the degree, betweenness centrality (BC), and closeness centrality (CC) of the nodes. The key active ingredients were screened based on their degree values with median BC and CC values serving as the threshold.

### GO functional analysis and KEGG enrichment analysis

The Bioconductor bioinformatics package (https://www.bioconductor.org/) was applied to convert gene names into gene IDs in the R 4.2.1 software. GO functional analysis and KEGG pathway enrichment analysis were then performed with a P-value of less than 0.05. The entries with the top 20 P values were chosen for generating entry maps and bubble maps.

### Molecular docking validation

Molecular docking was performed between the top five core active ingredients and the top-ranked core targets associated with the validated pathways to validate the target prediction results. The TCMSP platform provided the "mol2" format of the key active ingredient, which was then converted to the "pdbqt" format using Autodock Tools 1.5.6 software after determining the root of the ligand and selecting the twistable key and checking the charge. The PDB Protein Database was used to obtain the 3D structure of the core target, which was imported into Autodock Tools 1.5.6 software. Small molecule ligands and water molecules were removed using PyMoL 2.3.1 software (www.pymol.org/). Hydrogen was added, and the total charge was calculated before saving it in "pdbqt" format. Autogrid4 and autodock4 plug-ins were used for spatial localization and molecular docking, respectively. The "Local Search Parameters" algorithm was used for docking operations, and "Docking Parameters" were set. Binding energy ≤ -5.0 kJ/mol was considered to have a strong binding force, and the number of hydrogen bonds between the receptor and target protein was taken into account while interpreting the results^10^.

### Experimental drugs

The experimental drugs used in this study were the Chinese herbal medicine vinegar SR and vinegar CR, obtained from Jinhua, Zhejiang Province, and purchased from Shenzhen Huahui Pharmaceutical Co.

### Cells

The human prostate cancer cell line PC3, with cell identification number 20160628-01, was obtained from the Beijing Beina Chuanglian Institute of Biotechnology. The cells were cultured in F12K medium supplemented with 10% fetal bovine serum and 1% penicillin and streptomycin, and maintained at 37 °C with 5% CO_2_.

### Main reagents

The following reagents were used in this study: CCK-8 (BS350B) purchased from Beijing Labgic Technology Co., Ltd.; reverse transcription kit (E047-01B) and real-time quantitative PCR kit (DP419) purchased from Tiangen Biotech Co. Shanghai Co., Ltd.; DMEM F12K medium (CTCC-002-007) and fetal bovine serum (FBS) purchased from GIBCO, Grand Island, NY, USA; Western Blot related reagents purchased from Beyotime Biotech lnc.; p-AKT(R22961) purchased from ZEN BIO; p-mTOR (Ab137133) purchased from Abcam; and cleaved CASP9(10380-1-AP) purchased from Proteintech.

### Main instruments

The following instruments were used in this study: electronic Balance from Tianjin Tianma Hengji Instrument Co., Ltd.; DSZ2000X fluorescence microscope from Zhongxian Hengye Co., Ltd., Beijing; DH-160ICO2 incubator from Santon Instruments & Equipment Co., Ltd., Shanghai; EIX808U enzyme standard analyzer from BioTek, USA; CytoFLEX flow cytometer from BECKMAN COULTER, USA; DYCZ-24DH electrophoresis instrument and DYY-7C transmembrane instrument from Liuyi Biotechnology Co., Ltd., Beijing; and ChemiScope 6100 chemiluminescence imaging system from Qinxiang Scientific Instruments Co., Ltd., Shanghai.

### Extract preparation of SR-CR drug pair

To prepare the extract, 50 g of SR and 50 g of CR were taken into a 2L distillation flask, and 800 mL of 70% ethanol was added. The flask was placed in a constant temperature water bath at 95 °C for 1 h for the first extraction. The liquid was then extracted, and 600 mL of 70% ethanol was added to the flask for a second extraction at a constant temperature water bath for 0.5 h. The extracts were mixed and concentrated using a rotary evaporator at 60 °C and 60 rpm until solids were precipitated. The resulting material was pre-cooled in a − 20 °C refrigerator and then further concentrated to a lyophilized powder using a vacuum freeze dryer. The obtained lyophilized powder weighed 12.55 g, yielding 12.55% (based on 100% concentration). The lyophilized powder was dissolved in ultrapure water to prepare a concentration of 8 mg/mL and filtered through a 0.22 um diameter microporous filter for bacteria before use.

### Cell culture

PC3 cells were cultured in a complete medium consisting of a 5 mL penicillin/streptomycin mixture, 50 mL fetal bovine serum, and 445 mL F12K medium. The cells were then incubated at 37 °C, 5% CO_2_, and saturated humidity in a cell culture incubator. The cells were grown in a monolayer attached to the wall, and those in the logarithmic growth phase were selected for subsequent experiments.

### CCK8 experiments

To conduct the CCK8 experiments, PC3 cells in the logarithmic growth stage were taken, and their density was adjusted. The cells were then inoculated into 96-well plates at a density of 5000 cells/well, with 100uL of complete culture solution containing cells. The plates were then incubated at 37 ℃, 5% CO_2_, and saturated humidity for 24 h. A concentration gradient of the SR-CR drug pair was created, ranging from 8 to 0 mg/mL (8 mg/mL, 4 mg/mL, 2 mg/mL, 1 mg/mL, 0.5 mg/mL, 0 mg/mL), with 3 replicate wells for each group. A blank control group without drugs and cells was set up. The 96-well plates were incubated in the cell culture incubator for 24, 48, and 72 h. The complete culture solution was mixed with CCK8 solution at a 9:1 ratio and added to each well at a constant temperature incubator for 2 h. Finally, the 96-well plates were removed and placed in the enzyme marker, and the absorbance at 450 nm was measured to calculate the cell proliferation rate, as the following equation: 

$$\mathrm{Cell\,\, proliferation\,\, rate }=\frac{{{\text{D}}}_{adminstration\,\, wells }- {{\text{D}}}_{blank \,\,empty}}{{\mathrm{ D}}_{control \,\,wells}- {{\text{D}}}_{blank \,\,empty}}\times 100\mathrm{\%},$$

which D is absorbance. The IC of the drug was calculated using GraphPad Prism 8.0 based on the administered concentration and cell proliferation rate_50_.

### qPCR experiments

To conduct the qPCR experiments, logarithmically grown PC3 cells were taken, and their cell density was adjusted to 100,000/well. The cells were then mixed with 2 mL of complete culture medium and inoculated in a 6-well plate. The plate was then incubated in a cell culture chamber at 37 ℃, 5% CO_2_, and saturated humidity for 24 h. Three replicate wells were set up for the experimental group, and three replicate wells were set up for the control group. The IC_50_ dosing concentration was added to the experimental group, while the control group received a complete culture medium. The cells were then incubated together for 24 h. Total RNA was extracted by the Trizol method, and its purity was determined by nucleic acid micro-detector. A_260_/A_280_ at 1.8–2.0 was considered high purity of RNA extraction. The reverse transcription kit was operated according to the kit instructions. The primer sequences are shown in Table 3.

**Table3:** Primer design.

Gene	Primer sequence (5′ to 3′)	Primer sequence (3′ to 5′)
AKT1	CAAGGTGATCCTGGTGAA	CGTGGGTCTGGAAAGAGT
mTOR	AAACCTCGTCACATTACCC	GCGAGTTCTTGCTATTCC
CASP9	CGAACTAACAGGCAAGCA	CCAAATCCTCCAGAACCA
GAPDH	ACAACTTTGGTATCGTGGAAGG	GCCATCACGCCACAGTTTC

### Western blot experiment

PC3 cells were cultured until they reached the logarithmic growth phase, and then the cell density was adjusted to 100,000/well and mixed with 2 mL of complete culture medium. The mixture was then inoculated into a 6-well plate and placed in a cell culture incubator for 24 h. Three replicate wells were set up for the experimental group and three replicate wells for the control group. The experimental group was treated with the above IC_50_ dosing concentration for 48 h, while the control group was treated with a complete culture medium. After incubation together for 48 h, cell lysate was added to each well at 200 uL. The cells were scraped off and loaded into 1.5 mLEP tubes, and centrifuged at 12 000 r/min at 4 ℃ for 15 min. The supernatant was extracted from the total protein, and the protein concentration of each group was determined using the BCA method. Gel electrophoresis, membrane transfer, and closure were performed, followed by incubation with the corresponding primary antibody (1:500–5000) and secondary antibody (1:10,000). The relative protein expression was analyzed by ImageJ software and expressed as the gray value of the target protein/grey value of internal reference.

### Flow cytometry

PC3 cells in the logarithmic growth phase were taken, and the cell concentration was adjusted to 100,000 cells/well and then inoculated into 6-well plates. After 24 h, three replicate wells were set up for the control and experimental groups, where the control group was treated with a drug-free medium, and the experimental group was treated with the IC_50_ dosing concentration of SR-CR drug pair for 48 h. After treatment, the cells were washed twice with pre-chilled PBS, digested with EDTA-free trypsin, and collected by centrifugation at 4 ℃ for 5 min. The cells were then resuspended by adding 500 uL of 1 × Binding Buffer, 5uL Annexin V-FITC, and 5uL PI Staining Solution. Then, the cells were reacted for 10 min at room temperature, protected from light, and then detected using a flow cytometer.

### Statistical analysis

All statistical analyses were performed using SPSS 26.0 statistical software, with a significance level of ɑ = 0.05. Count data were reported as frequency (rate/composition ratio) and analyzed using the chi-square test. Measurement data were expressed as mean ± standard deviation, and GraphPad Prism 8.0 was used for graphing. Normality test and chi-square test were conducted prior to performing the independent samples t-test for comparison of the two groups. One-way ANOVA followed by the SNK method was used for comparison among multiple groups. In case where the variance was not homogeneous and did not follow a normal distribution, nonparametric tests were employed. A *P* value of ≤ 0.05 was considered statistically significant, and all *P* values were two-tailed.

## Discussion

Prostate cancer is a refractory malignancy, and the median time for cancer to progress from denervation to CRPC status is only about 19 months, and this state is irreversible^11–13^. Chinese medicine has made remarkable progress in the treatment of prostate cancer, especially in CRPC, and combined with surgery or chemotherapy. It has the effect of shrinking the tumor, lowering PSA, reducing recurrence, and prolonging its survival time^9,14,15^. SR-CR drug pair have been reported to have a positive effect on anti-tumor, although it is not clear^7,16,17^. In this study, the potential molecular mechanism of the herbal drug pair for the treatment of CRPC was elucidated based on network pharmacology and in vitro cellular experimental validation. The prediction of SR-CR drug pair for the treatment of CRPC revealed 7 active components, 59 shared targets, 1309 GO enrichment analyses, and 121 KEGG pathways. Molecular docking showed that the core active ingredients of SR-CR, beta-sitosterol, formononetin, wenjine, and bisdemethoxycurcumin, had good docking activity with the core targets AKT1, mTOR, and CASP9.

Several studies have reported significant tumor suppressive effects of the active components of SR-CR drug pair on CRPC. For example, hederagenin was reported to inhibit the proliferation, migration, and invasion of DU145 cells in a dose-dependent manner and to promote apoptosis^18^. Beta-sitosterol administration resulted in cell death while enhancing apoptosis^19^. Huang et al. found that formononetin greatly increased the proportion of early apoptotic cells in PC3 cells^20^. Bisdemethoxycurcumin may induce DNA damage through mobilization and redox cycling of genomic copper ions, thus inducing ROS generation and ultimately triggering apoptosis-like death in PC3, LNCaP, DU145, and C42B cells^21^. Overall, the SR-CR drug pair may be a potential therapeutic agent for the treatment of CRPC, and the molecular mechanisms underlying its efficacy may involve multiple targets and pathways. Further studies are needed to confirm these findings and optimize the therapeutic approach for CRPC treatment. Tian et al. used network pharmacological predictions to find that phytosterols such as Stigmasterol can exert resistance to CRPC through the prostate cancer signaling pathway^22^.

AKT is a serine/threonine kinase that plays a crucial role in regulating various cellular processes such as cell survival, proliferation, migration, metabolism, and angiogenesis^23,24^. Upon activation by growth signals, AKT phosphorylates downstream targets to regulate these processes. AKT1, in particular, has been implicated in multiple biological processes in tumors, including prostate cancer. Previous studies suggest that AKT isoforms, including AKT1 and AKT2, may play a role in prostate cancer progression from androgen-sensitive to hormone-refractory stages^25^. In animal models of CRPC, knockdown of AKT1/2 resulted in a significant reduction in tumor metastases, indicating that inhibition of AKT1 expression may improve survival prognosis for CRPC patients^26,27^.

mTOR is another serine/threonine kinase that regulates cellular metabolism and growth response to various cues. Its phosphorylated functional form, p-mTOR, has been implicated in many human diseases^28^. In CRPC, the mTOR pathway is frequently deregulated, and targeting mTOR has emerged as a promising therapeutic approach. Inhibition of mTOR and AR gene expression has been shown to effectively inhibit the growth of enzalutamide-resistant CRPC cells^29^. Additionally, mTOR inhibitors have been found to increase cellular GAS5 levels and inhibit growth in androgen-dependent and androgen-sensitive cell lines, but not in androgen-independent cell lines^30^.

The Caspase family of proteases plays a critical role in cell death and inflammatory response, and CASP3, CASP8, and CASP9 are among its members. In particular, CASP9 has been found to be enriched in the Prostate cancer pathway. Upon apoptosis induction, mitochondria release cytochrome c, which then binds to Apaf-1 and dATP to recruit and activate Caspase-9 (c-CASP9), leading to downstream caspase cleavage and initiation of the caspase cascade reaction. Therefore, CASP9 is a key regulator and initiator of apoptosis, and its induction may hold therapeutic potential in CRPC^31,32^.

The result of GO enrichment analysis showed that SR-CR drug pair mainly regulated PC3 cell proliferation and apoptosis by regulating the response to a steroid hormone, response to oxidative stress, protein serine/threonine/tyrosine kinase activity, and nuclear receptor activity and ligand-activated transcription factor activity. KEGG pathway enrichment revealed that cancer-related pathways were widely enriched, with the Prostate cancer pathway ranking first, indicating that SR-CR AKT1, mTOR, and CASP9 were all enriched in the Prostate cancer pathway. Additionally, molecular docking validation showed that active components of SR-CR, including beta-sitosterol, formononetin, wenjine, and bisdemethoxycurcumin, had good docking activity with the core targets of CRPC, AKT1, mTOR, and CASP9. AKT1, mTOR, and CASP9 were all located at the core of the Prostate cancer signaling pathway. SR-CR inhibited the Prostate cancer signaling pathway by downregulating the expression of AKT1 and mTOR, and upregulating CASP9, leading to apoptosis in prostate cancer cells.

It was found that SR-CR drug pair significantly inhibited the proliferation of PC3 cells and induced apoptosis, with early apoptosis being the predominant form. Decreased cell numbers, wrinkled morphology, and an increase in floating dead cells were also observed under the microscope. The result of the KEGG signaling pathway suggested that the inhibitory effect of SR-CR on PC3 cells might be mainly related to the Prostate cancer signaling pathway. The role of the Prostate cancer signaling pathway in the progression of prostate cancer is mainly to promote prostate cancer cell proliferation, enhance invasion and metastasis, and inhibit apoptosis. qPCR and Western Blot assay were used to investigate the effect of SR-CR on the expression of core targets. It was observed that SR-CR down-regulated the expression of AKT1 and mTOR genes and proteins and up-regulated the expression of CASP9 genes and proteins. The down-regulation of AKT1 and mTOR expression and the up-regulation of CASP9 expression implied that the conduction of the Prostate cancer signaling pathway is blocked, then this pathway-mediated proliferation was stalled and apoptosis was significantly increased. However, the differences in p-mTOR protein expression were not statistically significant due to the limited number of samples. Future experiments will include more samples for analysis and detect other target genes and protein expressions of this pathway to further validate the concept of this study.

## Conclusion

In summary, this study employed network pharmacology to investigate the disease targets and biological pathways associated with SR-CR drug pair in the treatment of CRPC. The study uncovered the impact of SR-CR drug pair on CRPC treatment by considering an “active ingredient-disease-shared target” Perspective. Using a PC3 cell model, the IC_50_ drug concentration was administered for 48 h to evaluate mRNA and protein expression of the key star molecules enriched in the Prostate cancer signaling pathway. The result demonstrated that one of the mechanisms of action of SR-CR drug pair in CRPC treatment is through inhibiting the Prostate cancer signaling pathway. However, the synergistic molecular mechanism of SR-CR drug pair on active ingredients requires further investigation to develop safe, effective, and economical natural anti-cancer drugs for clinical use and provide a new theoretical foundation for delaying the progression of CRPC.

## Data Availability

The data that support the findings of this study are available from the corresponding author upon reasonable request.
