# Author Correction: Exploring the mechanism of action of Sparganii Rhizoma-Curcumae Rhizoma for in treating castration-resistant prostate cancer: a network-based pharmacology and experimental validation study

**DOI:** 10.1038/s41598-024-55386-x

**Published:** 2024-03-01

**Authors:** Litong Wu, Haijun Chen, Yan Long, Junfeng Qiu, Xinjun Dai, Xujun You, Tiantian Li

**Affiliations:** 1 The First Clinical College of Traditional Chinese Medicine, Hunan University of Chinese Medicine, Changsha, 410208 People’s Republic of China; 2 School of Pharmaceutical Sciences, Hunan University of Medicine, Huaihua, 418000 People’s Republic of China; 3 Shenzhen Traditional Chinese Medicine Hospital, Shenzhen, 518033 People’s Republic of China; 4 Liuyang Hospital of Traditional Chinese Medicine Affiliated to Hunan University of Chinese Medicine, Changsha, 410300 People’s Republic of China; 5 Department of Andrology, Shenzhen Bao’an Traditional Chinese Medicine Hospital Group, Guangzhou University of Chinese Medicine, Shenzhen, 518100 People’s Republic of China; 6 Department of Otorhinolaryngology, Shenzhen Bao’an Traditional Chinese Medicine Hospital Group, Guangzhou University of Chinese Medicine, Shenzhen, 518100 People’s Republic of China

Correction to: *Scientific Reports* 10.1038/s41598-024-53699-5, published online 07 February 2024

The original version of this Article contained an error in the order of the author names, which was incorrectly given as Litong Wu, Yan Long, Junfeng Qiu, Xinjun Dai, Xujun You, Tiantian Li & Haijun Chen.

The original Article has been corrected.

